# Supplementary material for: Climate change, geography and trade agreements: A perspective of Asian bilateral trade
Source: PLoS One. 2025 Apr 25;20(4):e0320363. doi: 10.1371/journal.pone.0320363 (PMC12027022; doi:10.1371/journal.pone.0320363)
Supplement: Attachment — Submitted filename: LMR (1).docx [file pone.0320363.s001.docx]

***Review of the Manuscript "Climate Change, Geography and Trade Agreements: A Perspective of Asian Bilateral Trade"***

The manuscript presents a well-designed and comprehensive investigation of the simultaneous effects of geographic factors, trade agreements, and climate change on bilateral exports in Asian countries. The authors have employed a robust methodological approach, utilizing panel data from 2000 to 2020 and applying various econometric techniques, particularly the structural gravity model. The findings provide valuable insights into the importance of these factors in determining bilateral export levels.

**Introduction**:

The introduction section provides a strong foundation for the study. The authors have effectively highlighted the significance of geographic factors, climate change, and trade agreements in influencing international trade and bilateral exports (Section 1). The discussion on the role of natural and man-made geographic factors, as well as the potential impact of climate change and trade agreements, is well-articulated and sets the stage for the research objectives.

**Methodology**:

The methodology section is comprehensive and well-explained. The authors have developed a clear theoretical framework (Figure 1) and have utilized appropriate econometric techniques, such as the structural gravity model and the Heckman model, to estimate the impact of the various factors on bilateral exports (Sections 3.1 and 3.2). The choice of these methods is relevant and appropriate for addressing the research questions.

**Data**:

The data sources and variables used in the analysis are clearly described in Table 1. The authors have relied on reputable international organizations, such as the World Bank, CEPII, and UNCTAD, to collect the necessary data, which adds to the credibility of the study.

**Empirical** **Results**:

The empirical results are presented in a clear and systematic manner. The authors have conducted a thorough analysis, including the examination of the simultaneous impact of geographic factors, climate change, and trade agreements on bilateral exports (Tables 3-6). The findings demonstrate the importance of geographic factors, trade agreements, and the interaction between economic size and climate change in determining bilateral export levels.

**Robustness** **Checks**:

The authors have performed various robustness checks, including the use of alternative estimation methods, such as QREG, PPML, and 2SLS (Table 7). These checks help to ensure the reliability and consistency of the results.

**Conclusion and Recommendations:**

The conclusion section summarizes the key findings and provides valuable policy implications. The authors have suggested that countries should focus on improving natural and man-made geographic factors, signing trade agreements, and formulating policies to address the impact of climate change on bilateral exports (Section 6). The policy recommendations are well-aligned with the study's findings and provide a clear direction for policymakers.

**Recommendations:**

Based on the findings and conclusions of the study, the following minor recommendations can be made:

**1. Expanding the Scope of the Analysis:**

The authors acknowledge the limitations of the study and suggest that future research should incorporate additional factors, such as the impact of agglomeration, market access, and production costs, on bilateral exports (Section 6). Expanding the scope of the analysis can provide a more comprehensive understanding of the drivers of bilateral trade in Asian countries.

**2. Incorporating Detailed Geographic Factors:**

While the study examines the impact of various geographic factors, such as access to the ocean and common borders, the authors could consider incorporating more detailed geographic factors, such as the total land-use area by mountains, deserts, and seashores, to further enhance the understanding of their influence on bilateral exports (Section 6).

Overall, this is a well-designed and executed study that contributes to the understanding of the role of geographic factors, climate change, and trade agreements in determining bilateral exports in Asian countries. The authors have thoroughly addressed the research objectives and have provided a comprehensive analysis supported by appropriate empirical methods and robust findings.
